# TRAILblazing Strategies for Cancer Treatment

**DOI:** 10.3390/cancers11040456

**Published:** 2019-03-30

**Authors:** Anna-Laura Kretz, Anna Trauzold, Andreas Hillenbrand, Uwe Knippschild, Doris Henne-Bruns, Silvia von Karstedt, Johannes Lemke

**Affiliations:** 1 Department of General and Visceral Surgery, Ulm University Hospital, Albert-Einstein-Allee 23, 89081 Ulm, Germany; anna-laura.kretz@uni-ulm.de (A.-L.K.); andreas.hillenbrand@uniklinik-ulm.de (A.H.); uwe.knippschild@uniklinik-ulm.de (U.K.); doris.henne-bruns@uniklinik-ulm.de (D.H.-B.); 2 Institute for Experimental Cancer Research, University of Kiel, 24105 Kiel, Germany; atrauzold@email.uni-kiel.de; 3 Clinic for General Surgery, Visceral, Thoracic, Transplantation and Pediatric Surgery, University Hospital Schleswig-Holstein, 24105 Kiel, Germany; 4 Department of Translational Genomics, University Hospital Cologne, Weyertal 115b, 50931 Cologne, Germany; s.vonkarstedt@uni-koeln.de; 5 Cologne Excellence Cluster on Cellular Stress Response in Aging-Associated Diseases (CECAD), University of Cologne, Joseph-Stelzmann Straße 26, 50931 Cologne, Germany

**Keywords:** TRAIL signalling, TRAIL sensitising, TRAIL-induced apoptosis, TRAIL in cancer

## Abstract

In the late 1990s, tumor necrosis factor (TNF)-related apoptosis-inducing ligand (TRAIL), a member of the TNF-family, started receiving much attention for its potential in cancer therapy, due to its capacity to induce apoptosis selectively in tumour cells in vivo. TRAIL binds to its membrane-bound death receptors TRAIL-R1 (DR4) and TRAIL-R2 (DR5) inducing the formation of a death-inducing signalling complex (DISC) thereby activating the apoptotic cascade. The ability of TRAIL to also induce apoptosis independently of p53 makes TRAIL a promising anticancer agent, especially in p53-mutated tumour entities. Thus, several so-called TRAIL receptor agonists (TRAs) were developed. Unfortunately, clinical testing of these TRAs did not reveal any significant anticancer activity, presumably due to inherent or acquired TRAIL resistance of most primary tumour cells. Since the potential power of TRAIL-based therapies still lies in TRAIL’s explicit cancer cell-selectivity, a desirable approach going forward for TRAIL-based cancer therapy is the identification of substances that sensitise tumour cells for TRAIL-induced apoptosis while sparing normal cells. Numerous of such TRAIL-sensitising strategies have been identified within the last decades. However, many of these approaches have not been verified in animal models, and therefore potential toxicity of these approaches has not been taken into consideration. Here, we critically summarise and discuss the status quo of TRAIL signalling in cancer cells and strategies to force tumour cells into undergoing apoptosis triggered by TRAIL as a cancer therapeutic approach. Moreover, we provide an overview and outlook on innovative and promising future TRAIL-based therapeutic strategies.

## 1. Introduction

Pharmacotherapy for cancer uses one or more chemotherapeutic drugs with either a curative aim or to prolong life and manage symptoms. Chemotherapy targets rapidly dividing cells to slow or stop their growth, conversely not distinguishing between transformed and non-transformed cells which leads to the commonly observed strong side effects in most scenarios. The last decade has seen the development and testing of targeted therapies such as small-molecule inhibitors or monoclonal antibodies. The clinical use of a substance with the ability to selectively kill tumour cells while sparing healthy cells thereby reducing severe side effects is urgently needed in pharmacological cancer therapy. This “silver bullet” against cancer seemed close at hand in 1995 when tumor necrosis factor (TNF)-related apoptosis-inducing ligand (TRAIL/Apo2L) was introduced [1,2] and later shown to selectively induce apoptosis in tumour cells in vivo [3,4]. After much testing, this selectivity holds to this day, however, its efficacy to induce significant levels of apoptosis in some resistant cancers will have to be helped through additional means.

## 2. The TRAIL System

TRAIL is a type II transmembrane protein with apoptosis-inducing capabilities, which belongs to the TNF-superfamily (TNF-SF) [1,2]. In humans, apoptosis is induced by binding of TRAIL to the death receptors (DR) TRAIL-R1/DR4 (also called APO-2 or TNFRSF10A) [5] and TRAIL-R2/DR5 (TNFRSF10B, TRICK2, KILLER) [6]. TRAIL is active as a trimer, analogous to other TNF-SF members, and consequently binds three receptor molecules [7,8]. Besides TRAIL-R1 and TRAIL-R2, TRAIL engages TRAIL-R3 (DcR1, TNFRSF10C, TRID, LIT) and TRAIL-R4 (DcR2, DcR2, TNFRSF10D, TRUNDD) also designated as decoy receptors (DcR), and the soluble receptor osteoprotegerin (OPG, TNFRSF11B). These latter three receptors are thought to potentially negatively regulate apoptosis through TRAIL-R1 and -R2 by scavenging TRAIL [9,10,11,12,13,14]. TRAIL-R1 and TRAIL-R2 mediate apoptosis via a cytoplasmic death domain (DD), which is missing in TRAIL-R3 and is truncated in TRAIL-R4 [6,12,15,16,17]. In a physiological setting, the affinity of TRAIL to OPG is notably weaker compared to the membrane-bound receptors suggesting a subordinate role of the soluble receptor in TRAIL signalling [18,19]. Noteworthy, when being presented at low concentrations and under physiological conditions, TRAIL favours interaction with TRAIL-R2 [20]. Eventually, TRAIL-R2 was presumed as the preferential inducer of apoptosis. Though, this assumption has been debunked since, for instance, in lymphocytic leukaemia and a variety of pancreatic cancer cells, apoptosis is induced via TRAIL-R1 although TRAIL-R2 is present and functioning. Consequently, the precedence for one receptor seems to be tumour entity-dependent [21,22,23,24]. 

In mice, only one death-inducing TRAIL-R is expressed, mTRAIL-R (MK) sharing 43% and 49% sequence homology with human TRAIL-R1 and TRAIL-R2, respectively [25]. The murine decoy counterparts mDcTRAIL-R1 (TNFRS23) and mDcTRAIL-R2 (TNFRS22) lack a DD and differ widely from the amino acid sequence of human TRAIL-R3 and -R4 [26].

Despite a research history of more than 20 years, it is still puzzling why humans have evolved to express two death-inducing TRAIL-Rs [5,27]. In addition, TRAIl-R2 is expressed as short and long splice variant with a difference of an additional 29 amino acid stretch present in the extracellular domain of the long TRAIL-R2 isoform. However, a higher incidence of expression of the long isoform is described, whereas the ratio of the variants differs tissue-dependently [28]. 

## 3. The Apoptotic ‘TRAIL’

**Figure 1:**
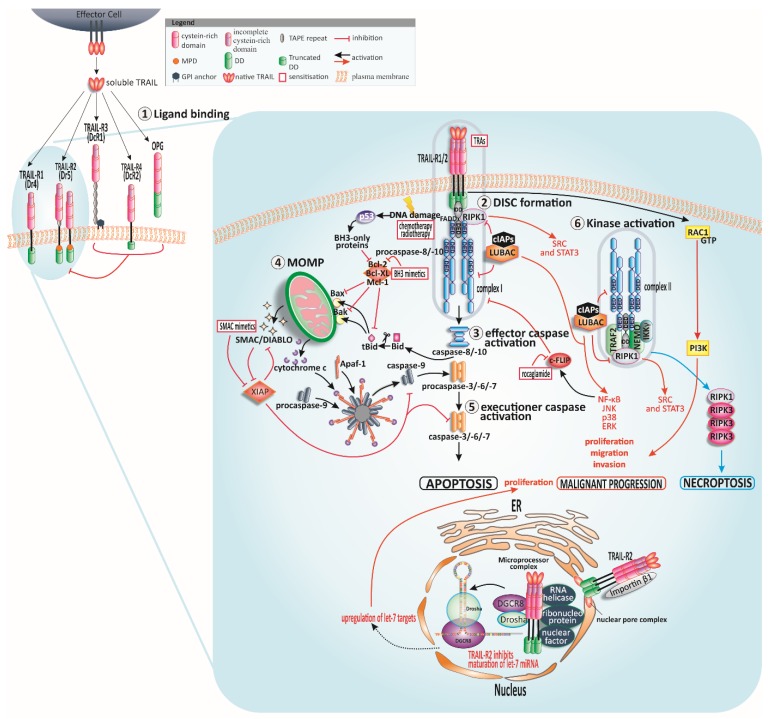
TRAIL signalling and sensitisation. Human tumour-necrosis-factor-related apoptosis-inducing ligand (TRAIL) can bind to four membrane-bound TRAIL receptors (TRAIL-Rs) and one soluble receptor ①. TRAIL-R1 and TRAIL-R2 can trigger apoptotic signals while the other receptors might serve as decoys and regulate apoptosis negatively. Cysteine-rich domains of the receptors are crucial for ligand binding. Following TRAIL binding to TRAIL-R1 or TRAIL-R2, the death-inducing signalling complex (DISC) is assembled ②. In type I cells, the signal of DISC-released caspase-8 is sufficient for activation of further downstream caspases and apoptosis induction ③, whereas the DISC signal is amplified via mitochondria in type II cells ④. Cleaved and thereby truncated Bid (tBid) activates the mitochondria-associated B-cell CLL/lymphoma 2 (Bcl-2)-family members Bcl-2-associated X protein (Bax) and Bcl-2 antagonist or killer (Bak) resulting in mitochondrial outer membrane permeabilisation (MOMP) and finally cytochrome c and second mitochondrial activator of caspases/direct inhibitor of apoptosis-binding protein with low isoelectric point (pI) (SMAC/DIABLO) release. The apoptosome comprising apoptotic protease activating factor-1 (Apaf-1), cytochrome c, and caspase-9, presents the activation platform for caspase-9 which leads eventually to the activation of executioner caspases ⑤. p53 is activated in response to stress signals such as DNA damage and promotes Bcl-2 homology (BH)3-only proteins resulting in MOMP. TRAIL-receptor-interaction can provoke the formation of a second cytosolic complex ⑥, retaining Fas-associated protein with death domain (FADD) and caspase-8 and recruiting receptor-interacting serine/threonine-protein kinase 1 (RIPK1), TNF receptor-associated factor 2 (TRAF2), and nuclear factor kappa-light-chain-enhancer of activated B cells (NF-κB) essential modifier (NEMO) [38,39,40]. TRAF2 recruits cellular inhibitor of apoptosis protein 1/2 (cIAP1/2) which in turn trigger the ubiquitination of RIPK1 and therefore recruitment of linear ubiquitin chain assembly complex (LUBAC) [41]. LUBAC poly-ubiquitinylates RIPK1. RIPK1 is the stimulus for tyrosine-protein kinase Src and signal transducer and activator of transcription 3 (STAT3) promoting migration and invasion [42]. Complex I and complex II induce NF-κB, p38 mitogen-activated protein kinase (p38 MAPK), c-JUN N-terminal kinase (JNK) and extracellular signal-regulated kinase (ERK) pathways. LUBAC is present in both complexes, responsible for caspase-8 activation and recruitment of the inhibitor of κB (IκB) kinase (IKK) complex, and consequently activation of NF-κB [41]. In case of blocked caspase-8, the necrosome is formed by the interaction of RIPK1 and RIPK3. Independently of FADD and complex I and II, the membrane-proximal domain (MPD) of TRAIL-R2 can activate Ras-related C3 botulinum toxin substrate 1 (Rac1) to promote migration and invasion [43]. TRAIL-R2 can also occur in the nucleus where it interacts with ribonucleoprotein complexes involved in the maturation of microRNAs (miRNAs) of the let-7 family. These miRNAs interact with and constrain mRNAs of several regulators of mitogenic pathways such as Ras and c-Myc thereby encouraging proliferation of tumour cells [44]. Further abbreviations: glycosylphosphatidylinositol (GPI), osteoprotegerin (OPG), death domain (DD).

The induction of apoptosis can be subdivided into either intrinsic apoptosis, triggered by p53 in response to cellular injuries [29] or extrinsic apoptosis induced upon death ligand binding to a death receptor. Caspases orchestrate both apoptosis pathways via cleavage of target proteins (reviewed in [30,31,32,33]). Conventional chemotherapy triggers intrinsic apoptosis through p53 in response to cellular damage. Nevertheless, many tumour entities contain mutations in p53 leading to its inactivation and failure of chemotherapy. In stark contrast to this, TRAIL-induced apoptosis remains possible in numerous cases despite non-functional p53 [34,35,36,37]. In Figure 1, the TRAIL-induced apoptotic signalling is illustrated. 

### 3.1. TRAIL-Induced Extrinsic Apoptosis

TRAIL binding to TRAIL-R1 and TRAIL-R2, respectively, initiates extrinsic apoptosis through receptor trimerisation and assembly of the death-inducing signalling complex (DISC) platform at their cytoplasmatic domains [45,46,47]. Caspase-8 and cellular FLICE-like IL-1β-converting enzyme-inhibitory protein long/short (c-FLIP_L/S_), are recruited to TRAIL-Rs via a homotypic interaction between the death domains (DDs) within the receptor and the adapter molecule Fas-associated death domain (FADD) [48,49]. The exact stoichiometric composition of the DISC unveiled three TRAIL-Rs recruiting one FADD molecule, its death effector domain (DED) in turn recruiting DED-only proteins (procaspase-8/procaspase-10 molecules or isoforms of c-FLIP [46,49,50]. The fact that procaspase-8 contains two DEDs has led to modelling of caspase-8 recruitment as a growing chain wherein merely the first procaspase-8 molecule directly binds to FADD [49,50]. Eventually, DISC formation allows procaspase-8/-10 dimerisation, its activation, and further activation of downstream caspases [13,48,49,50].

The caspase-8 homolog c-FLIP fulfils a particular function in regulating caspase-8 activity at the DISC [50,51,52,53,54,55]. Depending on the isoform, c-FLIP can act on caspase-8 in an antiapoptotic or proapoptotic manner [54]. An early, simpler concept of a negative regulation was based on the observation that c-FLIP could compete with caspase-8 for FADD binding [55,56,57]. Isoform-wise, c-FLIP long (c-FLIP_L_) is highly homologous to procaspase-8, but lacks a catalytic cysteine residue in the active site and therefore proteolytic activity. c-FLIP short (c-FLIP_S_) represents a shortened variant containing only tandem DEDs [58,59]. c-FLIP_S_ seems to perform anti-apoptotically, whereas c-FLIP_L_’s regulatory function is more complex [60]. Both splice variants, c-FLIP_S_ and c-FLIP_L_ can inhibit the activation of caspase-8 at the DISC when overexpressed [58,59,61]. Conversely, in an alternative scenario, c-FLIP_L_ and procaspase-8 can form an active heterodimer, which has been shown to promote apoptosis induction, but also to inhibit RIPK3-dependent necroptosis [62,63,64,65]. Recently, the prior model of procaspase-8 and c-FLIP competing for FADD banding has been revised at it was shown in vitro that c-FLIP recruitment to the DISC follows sequentially after an interaction of procaspase-8 and FADD has been established [54]. 

### 3.2. TRAIL-Induced Cross-Signalling to Mitochondria 

Soon after the discovery of TRAIL/TRAIL-Rs, it became evident that not all cells follow a similar linear pathway of TRAIL-induced extrinsic apoptosis. Some cells’ sensitivity depended on the ratio and expression levels of mitochondria-associated B-cell CLL/lymphoma 2 (Bcl-2) family proteins [66,67]. 

We now know that two cell types can be distinguished regarding their requirement to cross-signal to the mitochondrial apoptotic machinery [68]. In type I cells, DISC activation is sufficient to effectively trigger the full caspase cascade. Conversely, in type II cells, DISC activation does not result in efficient downstream caspase-3 activation and, hence, requires mitochondrial outer membrane permeabilisation (MOMP) to release the second mitochondrial activator of caspases/direct inhibitor of apoptosis-binding protein with low isoelectric point (pI) (SMAC/DIABLO) for counteracting protein X-linked inhibitor of apoptosis protein (XIAP) [68,69], a blocker of caspase-3, -7 and -9 [70,71,72]. Additionally, the level of XIAP is decisive in whether a cell undergoes type I or type II apoptosis [69]. MOMP is enabled by caspase-8-mediated cleavage of the Bcl-2 homology (BH)3-only protein (Bid) generating truncated Bid (tBid) [73], which translocates to the mitochondria and eventually activates the mitochondrial cascade (intrinsic apoptosis) by generating an oligomerisation [74,75] of Bcl-2-associated X protein (Bax) and Bcl-2 antagonist or killer (Bak) [73,76]. These Bcl-2 proteins permeabilise the mitochondrial outer membrane (MOM) leading to the release of cytochrome c into the cytosol. The cytochrome c concentration in the cytosol stimulates protease activating factor-1 (Apaf-1), which in turn mediates the assemblage of the apoptosome, the intracellular activation platform for procaspase-9. Active procaspase-9 promotes further activation of caspases-3, -6, and -7, which eventually execute apoptosis [33,77]. 

### 3.3. Checkpoints for TRAIL-Induced Apoptosis

Maintenance of the balance between death receptor-induced cell death and survival in non-malignant cells is achieved by numerous manifold control instruments [78], although not yet fully described, an interesting recent publication has revealed that many adult tissues downregulate pro-apoptotic proteins after birth, making them highly resistant against apoptotic stimuli [79]. The expression of decoy receptors can prevent apoptosis induction [80,81,82]. At the DISC, the inhibitor of caspase-8, c-FLIP, is upregulated following the induction of nuclear factor kappa light chain enhancer of activated B cells (NF-κB), thereby blocking apoptosis [83,84]. In type II cells, MOMP can be prevented when Bax and Bak are converted to their inactive form by binding of Bcl-2, Bcl-xL or induced myeloid leukaemia cell differentiation protein (Mcl-1) [84]. The release of cytochrome c upon MOMP is accompanied by SMAC/DIABLO [33,85,86]. SMAC/DIABLO binds and inactivates XIAP enabling effective activation of the effector caspase cascade and execution of cell death [66,84]. 

## 4. On the TRAIL for Targeted Cancer Therapy

In 1999, two independent groups demonstrated tumour regression in xenografts after systemic treatment with recombinant variants of human TRAIL (rhTRAIL) [3,4]. The consistent tumour cell-selective, apoptosis-inducing capabilities of TRAIL in preclinical research encouraged the development of clinical TRAIL-R agonists (TRAs). Two sets of TRAs used for clinical testing can be distinguished: (i) recombinant forms of human TRAIL and (ii) agonistic antibodies targeting TRAIL-R1 or TRAIL-R2 [14,87,88]. However, in order to achieve higher killing activity and considering the tissue-dependent preference of one specific TRAIL-R to induce apoptosis, an active agent targeting both apoptosis-inducing TRAIL-Rs is preferable [21,22,23,24].

### 4.1. TRAs in Clinical Studies—Can Failure Still Lead to Success?

**Figure 2:**
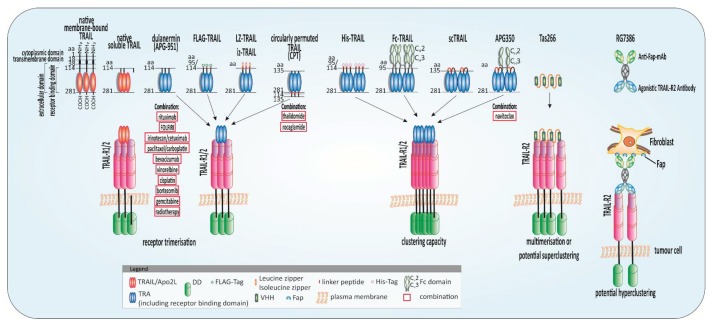
Highly potent TRAIL receptor agonists (TRAs) and combinatorial approaches. Schematic illustration of native tumour-necrosis-factor-related apoptosis-inducing ligand (TRAIL) and potent TRAs evaluated in clinical studies or with promising results in preclinical experiments and their combination with anticancer approaches: native membrane-bound TRAIL is a protein of 281 amino acids (aa) comprising a cytoplasmic domain (aa 1–17), a transmembrane domain (aa 18–38), and an extracellular domain (aa 39–281). The receptor-binding domain (aa 114–281) is located in the extracellular portion [1] which is cleaved from the cell surface to form bell-shaped homo-trimers. Recombinant human TRAIL/Apo2L (dulanermin) is based on the amino acids 114–281 of the extracellular portion of TRAIL [4]. The patients’ benefit in clinical trials was merely marginal even combined with chemotherapeutic regimens such as FOLFIRI (folinic acid, fluorouracil, irinotecan) [93,94]. Tags at the N-terminus of the extracellular domain of TRAIL such as FLAG and poly-histidine (His) aid purification of the ligand and leucine zipper (LZ) and isoleucine zipper (iz) stabilize the trimers [3,120]. Circularly permuted TRAIL (CPT) consists of the N-terminal amino acids 121–135 linked to the C-terminal amino acids 135-281 of TRAIL via a flexible linker [121]. To achieve the fragment crystallizable region (Fc)-TRAIL fusion protein, the Fc portion of human immunoglobulin G1 (IgG1) was fused to the N-terminus of human TRAIL (aa 95 to 281) [122]. Single-chain TRAIL (scTRAIL) is assembled by 3 extracellular domains of TRAIL that are covalently connected by 2 short peptide sequences [123]. APG350 was developed by the fusion of scTRAIL and the Fc-part of a human IgG1 leading to six receptor binding sites per drug molecule [124]. TAS266 comprises four humanised high-affinity heavy chain domain (VHH) antibody fragments which enable clustering of four TRAIL-R2s [117]. The substance caused severe hepatoxicity in clinical phase I studies. The tetravalent fibroblast-activation protein (FAP)-TRAIL-R2 antibody RG7386, targets cancer-associated fibroblasts in the tumour stroma and TRAIL-R2 on tumour cells simultaneously thereby inducing higher-order clustering and apoptosis induction in vitro and animal models [125].

First hopes in targeting a death receptor for cancer therapy became disillusioning when fulminant toxicity occurred in clinical trials of phase I and phase II testing recombinant TNF-α [89,90]. The TNF-SF system gained attention again when non-toxic and tumour cell-selective killing could be observed using TRAIL instead. Currently, only one recombinant form of human TRAIL, dulanermin (APO2L.0, AMG-951) (Figure 2), has reached clinical trials. The substance is untagged and comprises amino acids 114–281 of the extracellular portion of TRAIL and binds both death-inducing TRAIL-Rs [4]. Clinical phase I studies established tolerability and safety of TRAIL without dose-limiting toxicity (DLT), either as single-agent or in combination with conventional chemotherapeutic drugs for advanced non-small-cell lung cancer (NSCLC), non-Hodgkin lymphoma, colorectal cancer, advanced cancers, and B-cell lymphoma [91,92,93,94,95,96,97]. A maximum tolerated dose (MTD) could not be reached in the dose escalation study [96]. Nonetheless, the antitumor effects from preclinical models could not be confirmed in a phase II study paired with paclitaxel, carboplatin, and bevacizumab in advanced NSCLC [98]. The efficacy of dulanermin was eminently limited by its serum half-life of only 30–60 min restricting its ability to reach and kill cancer cells [99], and to achieve a steady-state concentration without continuous parenteral administration. Due to the weak response to dulanermin, it was not practicable to find biomarkers to predict the activity of the substance and patients’ response [100]. Following this initial failure, in 2017 the results of a phase III study of dulanermin combined with the semi-synthetic vinca-alkaloid vinorelbine [101] and the platinum-based cisplatin [102] for patients with NSCLC have been published. The objective response rate (ORR) was 46.78% in the treated group versus 30.00% in the placebo group. The progression-free survival (PFS) could be doubled in the treatment arm, however, a significant effect on the overall survival (OS) was not detectable [103]. It is thus doubtful, whether the response of the treatment was generated from the combination of dulanermin with the cytostatics or merely from the established drugs alone.

Agonistic TRAIL-R-specific antibodies also entered clinical trials. These are mapatumumab targeting TRAIL-R1 and conatumumab, lexatumumab, tigatuzumab, drozitumab, and LBY-135 which are all directed against TRAIL-R2 [87]. An advantage of these antibodies is their prolonged half-life in serum, usually prolonged from days to weeks. Just like dulanermin, these agonistic antibodies achieved favourable results in preclinical experiments [104,105] and were well tolerable and safe in first launched in-human studies [96,106,107,108,109,110,111]. Despite improved half-lives, their anticancer activities have been very limited. 

Given its positive preclinical performance, TRAIL has been downgraded from a ‘non-plus ultra’ agent for cancer treatment to a ‘plus ultra’ agent. Noteworthy, anti-TRAIL-R antibodies have been primarily evaluated in solid tumours. Blood perfusion of solid tumours is significantly restricted to provide a hypoxic, pro-tumorigenic surrounding. Poor perfusion within solid tumours might have hampered TRAIL’s efficacy in these entities [112]. Moreover, solid tumours are often protected by a complex immune- and non-immune microenvironment [113]. Another shortcoming of TRAIL-R targeting antibodies might lie in their Y-structure providing only two TRAIL-R-binding epitopes whereas efficient TRAIL-R-triggering requires cross-linking of receptor trimers [114,115]. Although, TRAIL-R-targeting antibodies failed in most patients, a small percentage responded to monotherapy. Therefore, predictive biomarkers would offer the potential to identify patients who might benefit. In 2007, a first such biomarker was published. The study found polypeptide N-acetylgalactosaminyltransferase 14 (*GALNT14*) expression to correlate with the cells’ sensitivity to TRAIL [116]. However, this biomarker of response could not be confirmed in patients, so far.

Nevertheless, high demand for targeting strategies while sparing healthy cells led to further development of TRAIL-R-targeting antibodies. TAS266 (Figure 2), a TRAIL-R2-specific, tetravalent nanobody was evaluated in clinical studies [117]. The small size of nanobodies is advantageous for distribution and tumour targeting. Moreover, nanobodies are highly stable, soluble, and specific and can be easily cloned [118]. TAS266 comprises four humanised high-affinity heavy chain domain (VHH) antibody fragments which enable clustering of four TRAIL-R2s [117]. The substance is an example for the pitfalls which come with excessively potent molecules since fulminant liver toxicity obliged the termination of the phase I clinical trial. Although the underlying mechanism causing the toxicity is not fully understood, the high potency of the molecule, potential immunogenicity and amplified TRAIL-R2 expression on liver cells may have caused an increased clustering of the receptors. In three patients suffering from the DLT pre-existing antibodies were detected able to bind to TAS266 [119]. 

Circularly permuted TRAIL (CPT) (Figure 2) is a novel form of recombinant human Apo2L/TRAIL with ongoing clinical evaluation for multiple myeloma (MM) and other hematologic cancers. It consists of the N-terminal amino acids 121–135 linked to the C-terminal amino acids 135–281 of TRAIL via a flexible linker. CPT’s pharmacokinetic is superior to wild-type TRAIL due to an enhanced stability and half-live [121]. In a single dose-escalation study adverse effects such as fever, fatigue, leucopenia, and vomiting remained below 10% incidence, and no immune responses were traceable [126]. To further evaluate the safety and efficacy of CPT as single agent, a phase II open-label study has been initiated. The monotherapy was well tolerated with 20–30% partially- or better-responding patients. No DLT and MTD could be observed [127]. A further dose-finding phase II study enrolled thalidomide-resistant relapsed refractory multiple myeloma (RRMM) patients and investigated the combination of CPT with the MM-approved drug thalidomide. The drug has been initially distributed under the trade name Contergan^®^ (Chemie-Grünenthal, Aachen, Germany) as sleeping pill, known from the Contergan disaster [128]. The ORR was 22%, however, treatment-related adverse effects (TRAE) occurred more often in the combinatorial treatment versus the single-agent treatment. The rate of higher-grade adverse effects had been certainly lower compared to the treatment with first-line therapy for MM such as bortezomib [129] demonstrating a comparatively low toxicity of the treatment regimen. An antitumour effect could be denoted, while no DLT occurred [130]. A phase II study for RRMM was conducted to compare the safety and efficacy of CPT plus thalidomide combined with dexamethasone (CPT + TD arm) and thalidomide plus dexamethasone (TD arm) [131]. The trial wanted to harness the antiproliferative effect of the glucocorticoid dexamethasone on melanoma cells [132]. The ORR of the CPT + TD arm was 38.3% compared to the ORR of the TD arm with 25%. Occurring TRAEs were in large part of the lower grades, whereas around 20% of patients suffered from severe side effects [131]. Currently, CPT presents the exception considering the antitumour potential amongst the TRAs. These findings provided the rationale for prospective confirmatory studies. 

ABBV-621 is designed for higher-order clustering of TRAIL-Rs by fusing an immunoglobulin G1 (IgG1)-fragment crystallizable region (Fc) portion to a single chain trimer of TRAIL subunits. In this way, six TRAIL-R binding sites are available upon dimerization of Fc portions. As with other TRAs, the preclinical data were promising. Apoptosis-inducing efficiency was observed in hematologic malignancies alone and in combination with the sensitisers navitoclax and venetoclax (see Section 5.4. BH3 and SMAC mimetics plus TRAIL) [133]. Currently, pre-treated patients are recruited for a dose-escalation study in solid tumours and hematologic malignancies using ABBV-621 (ClinicalTrials.gov Identifier: NCT03082209). The results are expected in February 2021.

Although CPT is the exception amongst TRAs boasting attributable anti-tumour action in clinical settings, a notable patient number showed no responses to the treatment. The main reason for the disappointing outcome of the majority of TRAs can be found in the inherent insensitivity of tumour cells to TRAIL monotherapy or acquired resistance [134,135,136]. To overcome this resistance, more profound understanding of the underlying mechanisms is vitally important. 

### 4.2. TRAIL Resistance Mechanisms in Cancer

One hallmark of cancer is an intrinsic or acquired resistance to apoptotic signals [137]. The TRAIL-induced apoptosis pathway provides manifold steps at which sensitivity can be modified starting with regulation of TRAIL-R levels and stretching all the way to mitochondrial anti-apoptotic proteins (Figure 1) [138,139,140]. While the big picture of TRAIL resistance is still not completely understood, numerous details and steps of the mechanism have been revealed [13,14,87].

#### 4.2.1. TRAIL Receptors 

At the receptor level, mutations in TRAIL-R1 and TRAIL-R2 [141,142,143], and expression of the decoy receptors TRAIL-R3 and TRAIL-R4 lacking the functional intracellular DD may play a regulatory role in TRAIL-induced apoptosis [9,16,82]. DcRs, particularly TRAIL-R4, might compete for TRAIL binding or may disrupt trimerisation of the death-inducing receptors -R1 and -R2 [9,144,145] when co-expressed on the same cell. Indeed, TRAIL-R4 was shown to directly interact with the death-inducing receptors directly thereby disrupting efficient DISC formation [82,145], and to signal alternative, tumour-promoting pathways such as NF-κB. Intriguingly, DcRs expressed by stromal cells have been demonstrated to negatively influence TRAIL sensitivity of tumour cells [146]. There is mounting evidencethat in contrast with its name, TRAIL seems to generate versatile effects, while enhanced tumour progression and metastasis could be observed upon treatment with exogenous TRAIL and activation of alternative pathways [147]. Moreover, in *Kirsten rat sarcoma (KRAS)*-mutated cancer upregulated TRAIL-R2 and endogenous TRAIL act in a tumour-supportive manner [43], a concept which does not seem to be limited to *KRAS*-mutated cancers and also engages tumour-favourable polarisation of immune cells [148].

Post-translational modifications of TRAIL-Rs have gained increasing interest over the last couple of years [149]. *O*-Glycosylation of TRAIL-R2 was found to control the cells’ sensitivity to TRAIL (described previously as a possible biomarker (see Section 4.1 above TRAs in Clinical Studies—Can Failure still lead to success?). A whole genome-profiling approach identified a correlation of increased *GALNT14* and TRAIL resistance. Cells could be sensitised to apoptosis when *GALNT14* was silenced [116]. In line with the idea that posttranslational modifications of the receptors have a considerable effect on TRAIL binding and killing, *N*-glycosylation of TRAIL-R1 increased its apoptotic efficacy [150,151].

Besides their membrane localisation, TRAIL-Rs have been observed also in intracellular compartments and in the nucleus [152,153]. The cellular localisation of TRAIL-Rs seems to play a growing role in TRAIL non-apoptotic signalling. Recent experiments revealed a correlation between increased levels of nuclear TRAIL-R2 and shortened patient survival, indicating a tumour-promoting role of the nuclear variant [44].

#### 4.2.2. The DISC

At the DISC level, FADD was shown to be indispensable for TRAIL-triggered apoptosis and loss of function led to resistance to TRAIL [48,154]. c-FLIP overexpression was detected in TRAIL-resistant cancer [155,156]. This antiapoptotic regulator and caspase-8 seem to be critical factors and unique molecules in death-receptor apoptosis signalling [48,55,139,157,158,159]. Recent discoveries identified a novel deubiquitylase of c-FLIP_L_, ubiquitin-specific peptidase 8 (USP8), directly interacting with the long isoform leading to its stabilisation and thereby inhibition of DR-induced apoptosis. Interestingly, increased levels of USP8 were found in melanoma and cervical cancer [160]. Changes in c-FLIP [155,161,162] and caspase-8 ratio [163] can trigger cell death-independent cellular responses via a cytosolic signalling complex termed complex II [164]. This complex II includes, besides the DISC components FADD and caspase-8, receptor-interacting protein kinase (RIPK1), TNF receptor-associated factor-2 (TRAF2) and NF-κB essential modifier (NEMO)/inhibitor of κB (IκB) kinase (IKK) [38,39,40]. TRAF2 recruits cellular inhibitor of apoptosis protein 1/2 (cIAP1/2) that in turn promote the ubiquitination of RIPK1 [165] thereby presenting the recruitment platform for linear ubiquitin chain assembly complex (LUBAC) [41]. LUBAC adds linear poly-ubiquitin chains to RIPK1 and completes tasks in complex I and complex II, enabling caspase-8 cleavage and IKK complex assembly, and accordingly activation of NF-κB [27,35,41,166,167,168,169,170,171,172]. Triggering the NF-κB pathway is one example of TRAIL’s non-canonical signalling routes occurring at the DISC level and conveying TRAIL resistance (Figure 1). Moreover, activation of mitogen-activated protein kinase (MAPK) [173,174] and tyrosine kinase Src and phosphoinositide 3-kinase (PI3K) pathways [175] through this complex II have been described [14]. 

#### 4.2.3. Bcl-2 Family

TRAIL resistance can also be attributed to expression patterns of the Bcl-2 family [13,87]: If cells undergo type II extrinsic apoptosis, death signals need the amplification via the mitochondrial pathway. Here, the ratio of the proapoptotic proteins Bax and Bak [176] and their antiapoptotic antagonists Bcl-2, Bcl-xL, and Mcl-1 [177,178,179,180,181,182] govern cell fate [78]. The mitochondrial release of SMAC/DIABLO ensuring activation of effector caspases, for example, is inhibited by Bcl-2 overexpression [183].

#### 4.2.4. IAPs

As indicated by their name, inhibitors of apoptosis proteins (IAPs) can block programmed cell death [184] and mediate TRAIL resistance [180,181,185,186,187] by regulating effector caspase activity such as caspase-3 or by inhibiting caspase-9 and apoptosome activity. An upregulation of IAPs has been described as a common feature of cancer [188,189,190]. The human IAP family comprises eight members so far, which have at least one baculovirus IAP repeat (BIR) domain in common: neuronal apoptosis inhibitory protein (NAIP; BIRC1), cIAP1, cIAP2 (BIRC2 and BIRC3), XIAP (BIRC4), survivin (BIRC5), BRUCE (Apollon; BIRC6), livin (BIRC7), and testis-specific IAP (Ts-IAP; BIRC8). Caspases of the apoptotic cascade are inhibited by XIAP, cIAP1, c-IAP2, and survivin [191,192]. 

#### 4.2.5. Autophagy—The Self-Consuming TRAIL

Besides its apoptosis-inducing capabilities, TRAIL is associated with autophagy. Autophagy (or autophagocytosis) is an essential cellular degradation mechanism to remove damaged or redundant proteins and cell organelles [193]. Since the 1950s, the mechanism has enthralled cell scientists. The cell biologist Yoshinori Ohsumi was rewarded with the 2016 Nobel Prize in Physiology or Medicine for the early description of the autophagy machinery [194], while other aspects are still obscure. The mechanism is involved in growth regulation and ageing processes and a diversity of other cellular functions such as differentiation and pathogen defence [195,196,197]. During periods of increased nutritional stress, the cellular components are recycled to maintain cell survival. Apoptosis and autophagy’s elements have various points of contact indicating the multifaceted cross-talk between the pathways. Usually, autophagy blocks apoptosis induction, and caspase activation in the apoptotic cascade shuts off autophagy signalling. One of the crucial controllers of autophagy is the mechanistic target of rapamycin (mTOR) kinase controlled by starvation, growth factors, and cellular stressors [197,198]. Upon DNA damage, p53 mediates the activation of AMP-activated protein kinase (AMPK) and thereby the blockage of mTOR and autophagy [199]. However, collected data over the last decade describes that p53 can also act in an autophagy-activating manner [200,201]. Beclin 1 (autophagy-related gene 6, ATG6) holds a vital role in the surveillance of autophagic actions [202,203] and the crosstalk between apoptosis and autophagy [198]. The Bcl-2 family members Bcl-2, Bcl-xL, and Mcl-1 are regulators of Beclin 1 by directly interacting with its BH3 domain. Noteworthy, the underlying mechanism of how these proteins either inhibit or stimulate autophagy and apoptosis is poorly understood [204]. 

Apoptosis and autophagy are both stimulated in response to metabolic stressors, however, in tumour cells, a different picture emerges, and the dialogue between autophagy and apoptosis is disrupted [193,205,206]. Generally, the nature of autophagy in cancer is bivalent, most likely describable as dynamic, demonstrated by either tumour-supportive or -suppressive actions [206,207,208]. Due to this interplay of autophagy and apoptosis, studies have been focused on the link between TRAIL resistance and autophagy. The connection between the mechanisms has become tangible when TRAIL has been shown to induce both, apoptosis and autophagy, in a series of cancer cell lines [209,210,211]. For cytoprotective TRAIL-induced autophagy, the activation of TRAF2- and RIPK1-mediated MAPK8/c-Jun N-terminal kinases (JNK) signalling [212] and AMPK signalling [213] was reported. In a nutshell, these results suggest that several pathways and regulators work together in a concerted manner to mediate TRAIL-induced apoptosis and autophagy. There are accumulating results in recent years identifying further regulators which maintain the crosslinking of apoptosis and autophagy. Notable, caspase-9 as such regulator facilitates the early events leading to autophagosome formation by complexing with ATG proteins thereby suppressing the apoptotic character of the caspase [214]. 

Autophagy controls the timing and efficiency of TRAIL-induced MOMP by the level of autophagy-releasing p53 upregulated modulator of apoptosis (PUMA, also known as Bcl-2-binding component 3) [215], a proapoptotic BH3-only Bcl-2 family member and downstream target of p53. MOMP is inefficient in cells with low PUMA levels, and some of these cells have been observed to recover and divide again whereas high PUMA levels led to MOMP quickly followed by apoptosis [216]. Most solid tumours exhibit areas permanently or transiently under hypoxic conditions resulting from abnormal vascularisation and blood flow [217]. The hypoxia intensifies mitochondrial autophagy in colorectal cancer cells. This loss of the cell organelles leads eventually to the scarcity of mitochondria-derived pro-apoptotic molecules such as SMAC thereby disrupting the transmission of TRAIL-mediated apoptotic signals in type II cells [218]. 

Recently, the accumulation of autophagic organelles in TRAIL-resistant cancer cell lines has been described. It was proven that this accretion led to TRAIL resistance in breast cancer cells that was accompanied by decreased surface expression of TRAIL-R1 and TRAIL-R2 [219]. The influence of autophagy on TRAIL-induced apoptosis in metastasis was just investigated by use of in vitro circulating tumour cells (CTCs) models for breast cancer which developed resistance to TRAIL with transition to a non-adherent state. This resistance was connected with downregulated surface levels of TRAIL-R2. The CTCs showed a fast autophagic flux detectable by an increase of autophagosome organelles where TRAIL-R2 was degraded [220]. The data obtained reveal a potential mechanism of how autophagy contributes to bypass TRAIL-induced apoptosis in CTCs. 

Taken together, TRAIL-induced apoptosis and autophagy are controlled by a plethora of pathways and often shared regulators that are not fully explored. This crosstalk gains in importance, and insights into this highly complex network in the period ahead contributes to novel treatment approaches in TRAIL-related cancer therapy including autophagy inhibitors such as beclin 1 inhibitors or inhibitors of PI3K. 

#### 4.2.6. Fractional Killing and Microenvironment

TRAIL resistance can occur during treatment cycles when only a fraction of cancer cells die due to cell cycle-imposed variations in pro- and anti-apoptotic protein levels. This enigmatic phenomenon has a non-genetic origin termed “fractional killing” [221]. A hypothesis for fractional killing by TRAIL is a caspase activation threshold determined by caspase inhibitory protein levels in cells at the time of TRAIL stimulation [222]. Eventually over time, monotherapy with TRAIL may select for survival of a resistant cell population and inefficiency of the therapy without any genetic predictors causing this process of natural selection. In keeping with this notion, cells surviving TRAIL-treatment within a population have been identified as the primary source for secreted cytokines such as interleukin 8 (IL-8) [223]. Moreover, overexpression of TRAIL-R1/R2, and engagement of an agonistic antibody with TRAIL-R2 can induce the release of inflammatory cytokines in an NF-κB-dependent manner [224]. The cancer microenvironment is permanently governed by these inflammatory factors described as a hallmark of cancer to protect the tumour and drive its progression [148]. Monotherapy with TRAIL and acquired resistance or resulting resistance from fractional killing may, therefore, promote the modulation of the tumour-promoting and -protecting microenvironment. Vice versa, the microenvironment seems to regulate TRAIL sensitivity of tumour cells via DcRs expressed on stromal cells [146].

## 5. Strategies to Regain TRAIL Sensitivity—The Bench-to-Bedside TRAIL

So far, most clinical studies evaluating TRAs have been discontinued due to insufficient anti-tumour effects. A fundamental reason is the intrinsic or acquired TRAIL resistance of cancer cells. Fortuitously, the various levels of regulation within the TRAIL-induced apoptotic pathway which can provoke resistance to apoptotic signals, offer equal opportunities to sensitise cancer cells to TRAIL-induced apoptosis [13,87]. Whilst mechanisms that mediate resistance to TRAIL in non-malignant cells are not entirely uncovered, it is questionable and unpredictable if TRAIL-sensitising strategies are limited to cancer cells or may also affect healthy cells. In cells that express both death-inducing TRAIL-Rs, heterocomplexation was observed [225], although it is still obscure whether this phenomenon has a unique signalling function. Nevertheless, it would be advantageous to target both, TRAIL-R1 and TRAIL-R2, in tumours considering the tissue-dependent preference of one receptor. In an effort to overcome TRAIL resistance, the ligand has been combined with other anti-cancer drugs or with nature-derived products to enhance cell sensitivity, respectively, and more potent TRAs have been developed. Several TRAs are presented in Figure 2. A variety of sensitising strategies can be found in the literature and have been reviewed in depth [13,14,87]. Unfortunately, many of these approaches had not been verified in animal models, and therefore possible toxicity has been ignored. Therefore, in the following section, only sensitising approaches with a high chance for quick and efficient clinical translation will be discussed.

### 5.1. Highly Potent TRAs

TRAIL engages three receptors, each at the interface between two of its cysteine-rich domains. The trimer is stabilised via a zinc atom bound by cysteines that is crucial for its biological activity [226,227]. TRAs lacking the critical central atom were poorly soluble and tended to aggregate, presumably conveying liver toxicity [228,229]. Aggregated TRAIL can evoke increased immunogenicity when epitopes are exposed that are usually hidden. An untagged variant including stoichiometric zinc did not provoke significant hepatotoxicity [228]. 

Tags at the N-terminus of the extracellular domain of TRAIL, such as FLAG™ (peptide sequence DYKDDDDK) and poly-histidine (His, His6-Tag) aid purification of the ligand. Leucine zipper (LZ) and isoleucine zipper (iz) stabilise TRAIL trimers thereby increasing their agonistic potential [3,120]. Since liver toxicity of His-TRAIL and antibody-crosslinked FLAG-TRAIL has been reported, safety concerns were noted for these variants and research on non-tagged variants has been intensified [120,228,230]. Presumably, these safety concerns are causative for untagged dulanermin entering clinical trials. A hepatoxicity of LZ- and iz-TRAIL, however, could be excluded and a safe in vivo application has been performed [3,120,231]. 

Due to their bivalent binding mode, agonistic TRAIL-R antibodies are not able to induce higher crosslinking of TRAIL-Rs leading to insufficient DISC activation and weak apoptotic signals compared to the tagged variants of TRAIL [114]. The crosslinking capacity is limited by the absence of adequate Fcγ receptors in the surrounding of cancer cells [114,232]. The aim is therefore to obtain an antibody multimerisation resulting in TRAIL-R-clustering on cancer cells and amplified apoptosis induction. For this reason, research into novel, stable TRAs suited for this purpose is pressing ahead [13,87,233]. A further effort to amplify the DISC signal can be the combined treatment with rhTRAIL and TRAIL-R-targeting antibodies binding to distinct receptor epitopes, thereby enhancing DISC formation and apoptosis induction [234].

Prolonged half-life and an increased tendency for oligomerisation was achieved by the fusion of TRAIL to the Fc portion of human IgG1 (Fc-TRAIL). Although the capacity for apoptosis induction was higher in vivo and in vitro, no liver toxicity was observed [122].

A recent development is a hexavalent TRA, designed by the fusion of two trimers of the extracellular part of TRAIL to the Fc portion of human IgG1 (APG350) thereby creating six receptor binding sites per drug molecule [124]. Recently, the superiority of APG350 over soluble TRAIL has been studied in pancreatic cancer in vitro and xenograft models [235]. Whilst Bcl-xL overexpression rendered cells resistant, sensitivity could be re-established when cells were additionally treated with the BH3 mimetic navitoclax (see Section 5.4 BH3 and SMAC mimetics plus TRAIL) [235,236]. Although APG350 showed encouraging preclinical results, the urgent need for predictive biomarkers and TRAIL sensitising strategies is highlighted through its evaluation in Bcl-xL expressing cells. The formation of higher-order complexes induced by APG350 is not sufficient to overcome blocked mitochondrial apoptosis. 

The tumour microenvironment is known to influence the therapy response negatively [113]. DcRs on stromal cells influence TRAIL resistance [146], and, in turn, as recently reported, a TRAIL-induced cancer secretome triggers a tumour-promoting microenvironment [148]. These facts illustrate the relevance of the tumour microenvironment when considering TRAIL-based therapies. The bispecific, tetravalent fibroblast-activation protein (FAP)-TRAIL-R2 antibody, RG7386, targets cancer-associated fibroblasts in the tumour stroma and TRAIL-R2 on tumour cells simultaneously thereby inducing higher-order clustering and apoptosis induction in vitro and animal models. The approach showed promise as monotherapy as well as in combination with chemotherapeutics [125].

### 5.2. Chemo- and Radiotherapy Plus TRAIL

Additive effects of standard chemotherapeutic agents such as gemcitabine, irinotecan, and cisplatin together with TRAIL have been extensively tested in preclinical models [237]. Furthermore, radiotherapy was proven to synergistically induce apoptosis in combination with TRAIL [238]. Modified receptor expression, enhanced DISC formation, and influence on pro- and anti-apoptotic protein expression are amongst the presumed mechanisms leading to the chemo- and radiotherapy-induced sensitisation. These findings laid the foundation for the combination with TRAs also in clinical studies, so far with marginal effects. Hitherto CPT is an exception, since the ORR of the combined treatment arm was increased in comparison to treatment with chemotherapy alone [131]. 

The 26S proteasome inhibitor bortezomib is used in MM care and is applied also in other malignancies including breast cancer, colon cancer, and prostate cancer [239]. The exact mode of action of bortezomib is revealed bit by bit. At the TRAIL-R level, a shift of the apoptotic signals from TRAIL-R1 to TRAIL-R2 by internalisation and degradation of TRAIL-R1 could be observed [240]. It also seems that a crosstalk of apoptosis and autophagy plays a central role [241,242,243]. TRAIL’s part in the regulation of the autophagosome formation is dependent on the expression of the ATG proteins ATG5, ATG7, and beclin-1. This TRAIL-induced ATG expression was weakened by JNK. In combination with bortezomib the activation of extracellular signal-regulated kinase (ERK) has been shown to induce autophagy [244]. Conversely, the inhibitory role of sustained activation of ERK has also been demonstrated. Bortezomib may block the autophagic flux by induction of ERK phosphorylation [243]. The evidence-based sensitisation capacity for TRAIL-induced apoptosis directed the substance to clinical trials combined with the TRAIL-R2 specific TRA mapatumumab. Again, an additive therapeutic advantage was not detectable mainly due to the failure of the antibody for to sufficiently crosslink TRAIL-Rs (see Section 5.1 Highly potent TRAs) [87].

### 5.3. Inhibition of Anti-Apoptotic c-FLIP in Combination with TRAIL

c-FLIP is an essential anti-apoptotic regulator and can suppress TRAIL-mediated apoptosis [245]. Coming back to the clinical studies investigating CPT (see Section 4.1 TRAs in Clinical Studies—Can Failure still lead to Success?), the therapy was efficient in 20–30% of the patients. The remaining 70% were nevertheless resistant to CPT. Analysing the reasons, elevated expression of c-FLIP in RRMM patients was striking [246] which may mediate resistance to CPT by inhibiting the DISC. Prior experiments already demonstrated the sensitisation of human melanoma cells to TRAIL by suppression of c-FLIP [245]. In a recent study, resistance to CPT was counteracted in MM in vitro and in vivo by downregulation of c-FLIP by rocaglamide (Figure 1) [246]. Rocaglamides are phytochemicals with cancer inhibiting abilities [247]. The natural product in combination with TRAIL also generated impactful in vitro action in additional cancer entities including renal carcinoma [248] and hepatocellular carcinoma (HCC) [249].

### 5.4. BH3 and SMAC Mimetics Plus TRAIL

The mitochondrial pathway of apoptosis is an important starting point for TRAIL sensitisation since high IAP expression correlates with drug resistance and tumour progression [250]. Small-molecule inhibitors have been designed which mimic the XIAP-binding site of SMAC thereby antagonising multiple members of the IAP family in the mitochondrial apoptosis cascade (Figure 1). Hitherto, the preclinical evidence [251,252] and first clinical studies [253,254] of these SMAC mimetics indicate a particular aptitude for anticancer therapy as a single agent or in combination with chemotherapeutic drugs. Their real capability as TRAIL sensitiser has already been analysed in a broad range of cancers [192]. Thus, the SMAC mimetic birinapant made it into a clinical trial in ovarian cancer in combination with the TRAIL-R2 agonist conatumumab. In the phase 1b study the tolerability of the combinatorial approach was proven [255]. Future studies will reflect the actual suitability of the substances as a treatment regimen. 

Bcl-2 family members govern cell fate in the mitochondrial apoptosis pathway. Overexpression of the anti-apoptotic relatives Bcl-2, Bcl-xL, and Mcl-1 mediates resistance to apoptosis signals and is a frequent feature among human cancers. To re-sensitise cells to apoptotic signals, BH3 mimetics have been established to conquer the anti-apoptotic Bcl-2 family members (Figure 1) [256]. Candidates that reached clinical phases are ABT-199 (venetoclax) directed against Bcl-2, and ABT-263 (navitoclax) antagonising Bcl-2 and Bcl-xL [257,258,259,260,261]. The treatment-related effects of BH3 mimetics were striking. Hence, an orally bioavailable form of venetoclax was approved in 2016 by the United States Food and Drug Administration (FDA) for chronic lymphocytic leukaemia (CLL) [262]. An effective synergism of navitoclax and venetoclax together with TRAIL was observed in vitro [263,264]. The clinical TRA candidate ABBV-621 induced apoptosis alone and enhanced in combination with navitoclax and venetoclax in haematological tumours [133]. According to these studies, SMAC mimetics, and BH3 mimetics might be a suitable class of sensitisers emerging from experimentation to overcome resistance to TRAIL-induced apoptosis in patient trials. 

## 6. Conclusions and Future Perspectives 

During the last decade, research into novel anti-cancer drugs has especially revolved around the discovery of specifically-targeted, side effect-reduced approaches. In this context, the discovery of the tumour selectiveness of apoptosis induction by TRAIL in the late 90s was thought to be a breakthrough and research into TRAIL-induced signalling pathways has been intensified. Following favourable preclinical results of tumour cell-selective apoptosis induction, recombinant forms of TRAIL and TRAIL-R-targeting antibodies have been evaluated in clinical trials, though with sobering outcome in most cases. The drawbacks have been outlined above as well as strategies to overcome TRAIL resistance. Novel, highly potent TRAs have been designed. By the use of high-throughput screenings (HTS), the identification and development of these TRAs can become more efficient. Although the TRAs with improved agonistic potential may finally boast the desired antitumor effect in clinical settings, overcoming TRAIL resistance remains a struggle ahead. There is a stable demand for highly-potent and adverse effect-reduced TRAIL sensitisers without losing sight of possible, fulminant adverse effects. For more appropriate pre-clinical testing, humanised and patient-derived preclinical mouse models are warranted. Moreover, predictive biomarkers are urgently needed to identify responding patients or to follow therapy response more precisely. To date, everything suggests that CPT will perform superior to conventional chemotherapy in haematological malignancies. In case that this approach can be validated for other tumour entities, and efficient sensitisers such as SMAC and BH3 mimetics can be clinically validated in combination, targeting this receptor/ligand system might just turn out to be a “TRAILblazer” in cancer therapy. 

## Abbreviations

AMPK	AMP-activated protein kinase
Apaf-1	protease activating factor-1
Apo2L	Apo-2 ligand
ATG	autophagy-related genes
Bak	Bcl-2 antagonist or killer
Bax	Bcl-2-associated X protein
Bcl-2	B-cell CLL/lymphoma 2
Bcl-xL	B-cell lymphoma-extra-large
BH	Bcl-2 homology
Bid	BH3-only protein
BIR	baculovirus IAP repeat
CLL	chronic lymphocytic leukemia
CTC	circulating tumour cells
c-FLIP	cellular (FADD-like IL-1β-converting enzyme)-inhibitory protein
c-FLIP_L_	c-FLIP large
c-FLIP_S_	c-FLIP short
cIAP	cellular inhibitor of apoptosis protein
CPT	circularly permuted TRAIL
DcR	decoy receptor
DD	death domain
DED	death effector domains
DISC	death-inducing signalling complex
DLT	dose-limiting toxicity
DR	death receptor
ERK	extracellular signal-regulated kinases
FADD	Fas-associated death domain
FAP	fibroblast-activation protein
Fc	fragment crystallizable region
FDA	United States Food and Drug Administration
FLICE	FADD-like IL-1β-converting enzyme
FOLFIRI	folinic acid, fluorouracil, irinotecan
GALNT14	polypeptide *N*-acetylgalactosaminyltransferase 14
GPI	glycosylphosphatidylinositol
HCC	hepatocellular carcinoma
HSA	human serum albumin
HTS	high-throughput screenings
IgG	Immunoglobulin G
IKK	inhibitor of κB kinase
IL	interleukin
iz	isoleucin
IκB	inhibitor of κB
JNK	c-Jun N-terminal kinases
KRAS	Kirsten rat sarcoma
LUBAC	linear ubiquitin chain assembly complex
LZ	leucine zipper
MAPK	mitogen-activated protein kinase
Mcl-1	myeloid leukaemia cell differentiation protein
miRNA	microRNA
MOM	mitochondrial outer membrane
MOMP	mitochondrial outer membrane permeabilization
MPD	membrane-proximal domain
MTD	maximum tolerated dose
mTOR	mechanistic target of rapamycin
NEMO	NF-κB essential modifier
NF-κB	nuclear factor kappa-light-chain-enhancer of activated B cells
NIAP	neuronal apoptosis inhibitory protein
NSCLC	non-small-cell lung cancer
OPG	osteoprotegerin
ORR	objective response rate
OS	overall survival
PFS	progression-free survival
pI	isoelectric point
PI3K	tyrosine kinase Src and phosphoinositide 3-kinase
PUMA	p53 upregulated modulator of apoptosis
Rac1	Ras-related C3 botulinum toxin substrate 1
rhTRAIL	recombinant human TRAIL
RIPK	receptor-interacting protein kinase
RRMM	relapsed refractory multiple myeloma
scTRAIL	single-chain TRAIL
SMAC/DIABLO	second mitochondrial activator of caspases/direct inhibitor of apoptosis-binding protein with low pI
SR	superfamily
tBID	truncated BH3-only protein
TD	thalidomide/dexamethasone
TNF	tumor necrosis factor
TNF-SF	TNF-superfamily
TRAF2	TNF receptor-associated factor-2
TRAE	treatment-related adverse events
TRAIL	TNF-related apoptosis-inducing ligand
TRAIL-R	TRAIL receptor
TRA	TRAIL receptor agonist
Ts-IAP	testis-specific IAP
USP8	ubiquitin-specific peptidase 8
VHH	high-affinity heavy chain domain
XIAP	X-linked inhibitor of apoptosis protein
